# The diagnostic performance of dental maturity for identification of the circumpubertal growth phases: a meta-analysis

**DOI:** 10.1186/2196-1042-14-8

**Published:** 2013-05-23

**Authors:** Giuseppe Perinetti, Graziela H Westphalen, Matteo Biasotto, Stefano Salgarello, Luca Contardo

**Affiliations:** Department of Medical, Surgical and Health Sciences, University of Trieste, Ospedale Maggiore, Piazza Ospitale 1, Trieste, 34129 Italy; Dipartimento di Specialità Medico-Chirurgiche, Scienze Radiologiche e Sanità Pubblica, Università di Brescia, Servizio di Odontostomatologia, P.le Spedali Civili 1, Brescia, 25123 Italy

## Abstract

The present meta-analysis initially evaluates the reliability of dental maturation in the identification of the circumpubertal growth phases, essentially for determining treatment timing in orthodontics. A literature survey was performed using the Medline, LILACS and SciELO databases, and the Cochrane Library (2000 to 2011). Studies of the correlation between dental and cervical vertebral maturation methods were considered. The mandibular canine, the first and second premolars, and the second molar were investigated. After the selection, six articles qualified for the final analysis. The overall correlation coefficients were all significant, ranging from 0.57 to 0.73. Five of these studies suggested the use of dental maturation as an indicator of the growth phase. However, the diagnostic performance analysis uncovered limited reliability only for the identification of the pre-pubertal growth phase. The determination of dental maturity for the assessment of treatment timing in orthodontics is not recommended.

**Electronic supplementary material:**

The online version of this article (doi:10.1186/2196-1042-14-8) contains supplementary material, which is available to authorized users.

## Review

### Introduction

It is well established that in growing subjects facial skeletal disharmonies, i.e. skeletal malocclusions, can be correctly treated by orthopaedic approaches. Such skeletal malocclusions include, for instance, transverse maxillary constrictions and mandibular deficiency or prognathism, which are relatively common features in certain ethnic populations [1, 2]. However, successful orthopaedic treatments in growing subjects are critically dependent on the skeletal maturation, i.e. the growth phase at which the treatment is performed [3, 4]. The important growth phases in such orthodontically treated subjects are circumpubertal, as the pre-pubertal, pubertal and post-pubertal growth phases [3–5], each of which is characterised by differential growth of the maxillary and mandibular basal bones [5–7].

As chronological age [5, 8] and dental emergence [8, 9] have been shown to be poorly related to skeletal maturation, at least during these circumpubertal growth phases, these parameters are known not to be reliable indicators for treatment timing [6, 10]. Therefore, over the last five decades, efforts have been carried out to find reliable and reproducible indicators of skeletal maturity in individual subjects [3, 5, 9, 11–13]. These indicators have included radiographic hand-and-wrist maturational stages [11] and cervical vertebral [3, 13] maturational (CVM) stages, along with non-invasive biomarkers in gingival crevicular fluid [14].

A further method is seen with dental maturity, which can be easily assessed through the evaluation of tooth formation [15], and which can be carried out on panoramic radiographs that are routinely used for different purposes, and with minimal irradiation to the patient. The degree of crown and root formation can also be assessed with minimal influence according to dimensional distortions that can be seen on panoramic radiographs [15]. In this regard, high correlations between dental and skeletal maturity have been reported by most of the investigations performed to date [12, 16, 17]. As a consequence, most of the studies have proposed that the staging of dental maturation is a reliable indicator of the individual skeletal maturity, which has major diagnostic implications [12, 16, 17].

However, a correlation analysis is not sufficient to reliably assess the diagnostic usefulness of dental maturation for the identification of the skeletal maturation phase in individual subjects. Thus, a dedicated analysis of diagnostic performance is needed. Such an analysis would need to include sensitivity, specificity, positive predictive values, and positive likelihood ratios (LHRs) [18, 19]. Interestingly, the only diagnostic performance study that has been performed to date reported little diagnostic value of dental maturation in the assessment of skeletal maturation, in spite of the high correlation coefficients retrieved [20].

A comprehensive meta-analysis regarding the relationship between dental and skeletal maturity is thus still missing, with a reappraisal of the diagnostic performances of previous investigations deemed necessary to definitively assess the diagnostic usefulness of dental maturation in the identification of skeletal maturity. Therefore, the present study was based on the appraisal of these missing diagnostic performance analyses in previous investigations, which were then used for the subsequent meta-analysis. In particular, studies on the maturation of the mandibular teeth in growing subjects who had never been orthodontically treated were considered.

### Materials and methods

#### Search strategy

The present meta-analysis follows the Preferred Reporting Items for Systematic Reviews and Meta-Analyses (PRISMA) [21] (see Additional file 1) and identifies all of the relevant studies in which possible correlations between dental and skeletal maturity in growing subjects at the circumpubertal growth phases were investigated. In particular, studies using the dental maturational staging according to Demirjian et al [15] (as individual teeth) and the reliable skeletal maturity assessment by the CVM method according to Hassel and Farman [13] or Baccetti et al [3] were considered. A literature survey was carried out through the following databases: Medline (Entrez PubMed, http://www.ncbi.nlm.nih.gov), Latin American and Caribbean Health Sciences (LILACS, http://lilacs.bvsalud.org), Scientific Electronic Library Online (SciELO, http://www.scielo.org), and the Cochrane Library (http://www.thecochranelibrary.com). The survey covered the period from 1 January 1995 to 30 November 2011, with no language restrictions. The following search algorithm was used in the databases, with the asterisk symbol (*) indicating truncation: ((dental age OR dental matur*) AND (skelet* matur* OR cervical vertebra* matur*)). For the search through the Cochrane Library, the whole Library (set at ‘search all text’) was screened with no restrictions as to the record status. Finally, a manual search was also performed by scoring the references within the studies examined and the titles of the papers published over the last 10 years in the following journals: *American Journal of Orthodontics and Dentofacial Orthopedics*, *The Angle Orthodontist*, *European Journal of Orthodontics*, *Progress in Orthodontics*, *Oral Radiology*, *Oral Surgery Oral Medicine Oral Pathology Oral Radiology*, and *World Journal of Orthodontics*.

The eligibility assessment and data collection processes were performed independently by two of the authors (GP and GHW). The data collection was carried without blinding to the authors. The intra-examiner reliability in the study selection process was assessed through the Cohen *k* test assuming a threshold value of 0.61 [22]. Conflicts were resolved by discussion of each article until a consensus was reached.

#### Study selection

**Figure 1:**
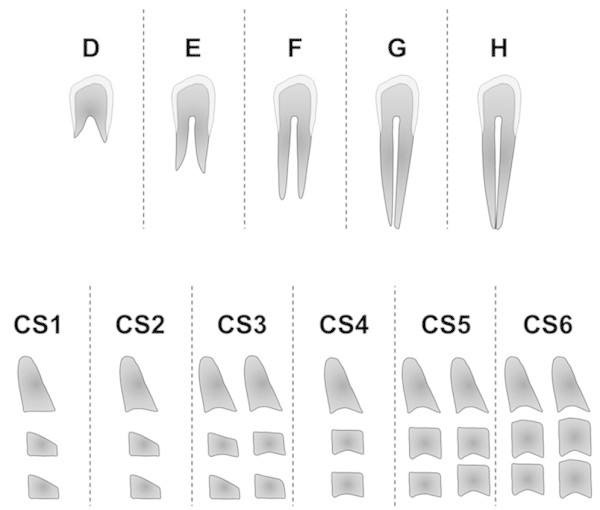
**Dental maturational stages and CVM stages.** Dental maturational stages according to Demirjian et al. [15] (stages D to H, upper) and CVM stages according to Hassel and Farman [13] or Baccetti et al. [3] (stages 1 to 6, lower). For the dental maturational stages, only the canine is shown for clarity. The CS1 and CS2 are pre-pubertal stages, CS3 and CS4 are pubertal stages, and CS5 and CS6 are post-pubertal stages (see text for details of the maturational staging).

The studies retrieved had to correlate dental and skeletal maturity in a cross-sectional design, with an analysis of the distribution of the different maturational staging of individual mandibular teeth across the skeletal maturational stages. The methodologies used had to comply with the following two requirements:

1. Use of the dental maturational method [15], comprising five stages (D to H) in the circumpubertal growth phases of the teeth investigated, as shown in Figure 1 and as briefly defined as follows:
  *Stage D*. When (1) the crown formation is complete down to the cemento-enamel junction; (2) the superior border of the pulp chamber in the uniradicular teeth has a definite curved form, with it being concave towards the cervical region; the projection of the pulp horns, if present, gives an outline shaped like the top of an umbrella; and (3) the beginning of root formation is seen, in the form of a spicule.
  *Stage E*. When (1) the walls of the pulp chamber form straight lines, the continuity of which is broken by the presence of the pulp horn, which is larger than in the previous stage, and (2) the root length is less than the crown height.
  *Stage F*. When (1) the walls of the pulp chamber form a more or less isosceles triangle, with the apex ending in a funnel shape, and (2) the root length is equal to or greater than the crown height.
  *Stage G*. When the walls of the root canal are parallel and its apical end is still partially open.
  *Stage H*. When (1) the apical end of the root canal is completely closed and (2) the periodontal membrane has a uniform width around the root and the apex. For mandibular molars, the distal root is considered for staging.
2. Use of the CVM method [3, 13], comprising six stages (CS), as shown in Figure 1 and as briefly defined as follows:
  *CS1*. When the lower borders of the second, third, and fourth vertebrae (C2, C3, and C4) are flat and the bodies of C3 and C4 are trapezoid in shape. CS1 occurs at least 2 years before the pubertal growth spurt.
  *CS2*. When only the lower border of C2 is concave and the bodies of C3 and C4 are trapezoid. CS2 occurs 1 year before the growth spurt.
  *CS3*. When the lower borders of both C2 and C3 have concavities and the bodies of C3 and C4 are either trapezoid or rectangular horizontal in shape. CS3 marks the ascending portion of the growth spurt.
  *CS4*. When the lower borders of C2 to C4 have concavities and the bodies of both C3 and C4 are rectangular horizontal. CS4 marks the descending portion of the growth spurt.
  *CS5*. When the lower borders of C2 to C4 have concavities and at least one of the bodies of C3 and C4 is square. CS5 occurs 1 year after the growth spurt.
  *CS6*. When the lower borders of C2 to C4 have concavities and at least one of the bodies of C3 and C4 is rectangular vertical. CS6 occurs at least 2 years after the growth spurt.

Studies that presented different maturity evaluation methods were excluded. Case reports, case series, reviews, and opinion articles were also excluded.

#### Data items

The following data items were collected: year of publication, ethnicity, investigated teeth, sample(s) size and age, results in terms of correlations between dental and cervical maturational stages, main diagnostic indications on specific dental stages in the identification of the skeletal maturation phase, and indications on the diagnostic usefulness of dental maturity in identification of skeletal maturity (Table 1). If a study also included the investigation of the maturity of any of the incisors, or of the first and third molars, they were not considered here because these teeth are usually fully developed in the pre-pubertal growth phase or are very late, such as for the third molars. Similarly, data regarding the maturity of the maxillary teeth were also excluded, as the presence of calcified structures that superimpose on these teeth renders the assessment of the maturational stages less reliable [17, 23].

#### Assessment of study quality and risk of bias in individual studies or across studies

The methodological soundness of each article was based on a quality evaluation method that followed pre-established characteristics that were modified from other methods reported previously [24, 25]. The following characteristics were used, along with the systematic scores that were assigned to the individual retrieved articles:

1. Adequacy of sample selection description based on three criteria: (1) age and gender; (2) ethnicity; (3) systemic health conditions, clearly excluding any growth or nutritional problem; (4) any further condition, i.e. use of drugs, that might alter dental and skeletal maturation; and (5) no history of orthodontic treatment (full description: 2 points; partial description: 1 point)
2. Method error analysis (full, for both dental and skeletal maturation assessment: 2 points; partial, for one assessment only: 1 point)
3. Adequacy of statistics (full analysis including performance diagnosis: 2 points; partial analysis without diagnostic performance: 1 point)
4. Previous estimate of sample size (1 point)
5. Blinding of measurements (1 point)

The quality of the studies was considered as follows:

1. Low: total score ≤ 3 points
2. Medium: total score > 4 and ≤ 6 points
3. High: total score > 6 points

Moreover, the PRISMA statements [21] for the assessment of risk of bias of individual studies have been considered here. According to these statements, the following items should be used: (1) concealment of randomisation, (2) clinical trial stopped early, (3) patients blinded, (4) healthcare providers blinded, (5) data collectors blinded, and (6) outcome assessors blinded. However, according to the designs of the studies considered here, only the blinding of data collectors and outcome assessors are applicable, and this was thus included in the item ‘blinding of measurement’ in the quality analysis.

**Table 1:** **Summarised data of the six studies included in the meta-analysis**

Study	Ethnicity	Investigated teeth	Sample(s) size and mean age(s) in years (range or SD)	Correlations between dental and cervical maturational stages	Main diagnostic indications on specific dental maturational stages in the identification of the skeletal maturation phase	Diagnostic usefulness of dental maturity in identification of skeletal maturity
Başaran et al. [23]	Turkish	Canine	295 M, 12.93 ± 1.91	Strict correlations along with differential behaviour of the teeth among the sexes	Not reported	Yes, for all of the skeletal maturation phases
		First premolar				
		Second premolar	295 F, 12.93 ± 1.91			
		Second molar				
Chen et al. [28]	Chinese	Canine	134 M, 8-16	Statistically significant correlations along with differential behaviour of the teeth among the sexes	Stage G of the canine for males and stage F of the second molar for females might signify the beginning of the pubertal growth spurt in Chinese subjects	Yes, for the onset of the pubertal growth spurt
		First premolar				
		Second premolar	168 F, 8-16			
		Second molar				
Sukhia and Fida [31]	Pakistan	Canine	147 M, 7-17	Statistically significant correlations along with differential behaviour of the teeth among the sexes	Stage H of the first premolar in males and second molar stage G for females are mainly at CS3	Yes, for all of the skeletal maturation phases
		First premolar				
		Second premolar	233 F, 7-17			
		Second molar				
Różyło-Kalinowska et al. [30]	Polish	Canine	287 M, 6-17	Moderate but statistically significant correlations along with differential behaviour of the teeth among the sexes	Not reported	Yes, but only for an initial assessment
		First premolar				
		Second premolar	431 F, 6-17			
		Second molar				
Kumar et al. [29]	Indian	Second molar	137 M, 9-18	Large and highly significant correlations. Similarity between the sexes	Up to stage E is mainly at CS2. Stages F and G are mainly at CS3 and CS4, and stage H is mainly at CS5 and CS6	Yes, for all of the skeletal maturation phases
			163 F, 9-18			
Perinetti et al. [20]	Italian	Canine	146 M, 7-17	High correlation coefficients with little differences between sexes and teeth	Canine up to stage F and all the resting teeth up to stage E are mainly at CS1 and CS2. Second molar stage H is mainly CS5 or CS6	Little. Mainly for the pre-pubertal growth phase
		First premolar				
		Second premolar	208 F, 7-17			
		Second molar				

#### Primary outcome of interest

To establish the clinical performance of each dental maturation stage for the diagnosis of each CVM stage, positive LHRs were calculated [19]. Positive LHRs provide estimates of how much a given dental maturation stage changes the odds of having a given growth phase. Here, a positive LHR indicates that a subject who tests positive for any clinical parameter (i.e. any dental maturation stage) has a high probability of having the given condition that needs to be diagnosed (i.e. a given growth phase). The positive LHR incorporates both the sensitivity and the specificity of the test, and it provides a direct estimate of how much a test result changes the odds of having a condition [19]. A threshold of a positive LHR of ≥10 [18] was considered for assessment of satisfactory reliability of any dental maturation stage for the identification of any of the growth phases. Therefore, positive LHRs, along with 95% confidence intervals (CIs), were calculated for each investigated tooth for the identification of the growth phases, which were defined as pre-pubertal (CS1 and CS2), pubertal (CS3 and CS4), and post-pubertal (CS5 and CS6). Dedicated statistical software was used to calculate the positive LHRs (MedCalc, version 12.0, MedCalc Software, Mariakerke, Belgium).

#### Secondary outcomes of interest

For each investigated tooth, the secondary outcomes of interest were the percentage distributions of the different maturational stages across the growth phases (by pooling the data for male and female subjects) and the correlation coefficients between the dental maturational stages and the CVM stages (both according to the male and female subjects, and by pooling the sexes).

#### Synthesis of results

The data were combined for meta-analysis using statistical software (Comprehensive Meta-Analysis software, Biostat Inc., Englewood, NJ, USA). Heterogeneity was assessed using the *χ*2-based Q-statistic method and *I*^2^ measurement, with significance set as *p* < 0.1; however, because of the moderate insensitivity of the Q statistic [26], only an *I*^2^ value ≥25% was considered associated to a significant heterogeneity among the studies [27]. Upon this analysis, a random effect model was used for all of the overall effect calculations [27]. Positive LHRs are reported as means and 95% CIs for both the point estimates and the overall effects. Percentage distributions of the different maturational stages across the growth phases are reported as means for the point estimates and as means and 95% CIs for the overall effects. As no relevant differences were seen between the sexes, these analyses are shown with pooling of the sexes. Forest plots for each meta-analysis present the correlation coefficients according to the sexes, point estimates (displayed as blocks), and CIs (displayed as lines).

### Results

#### Study search

**Figure 2:**
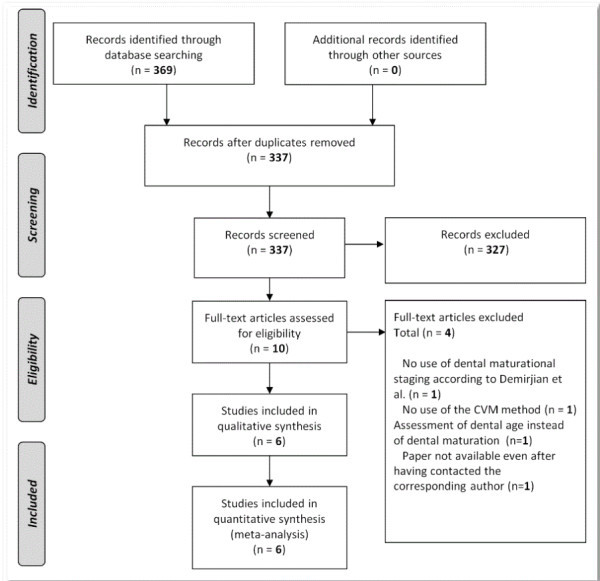
**Flow diagram of the search strategy.**

Of the 337 papers retrieved by the automatic and manual searches, six studies [20, 23, 28–31] (Table 1) were judged to be relevant according to the inclusion/exclusion criteria. All of these studies were included in the meta-analysis for all of the primary and secondary outcomes (Figure 2). Five studies [20, 23, 28, 30, 31] used the CVM method according to Baccetti et al [3], and one study [29] used the CVM method according to Hassel and Farman [13].

#### Study populations and main reported results and conclusions

The main features of the studies included are given in Table 1. All of the studies analysed different ethnic populations, including Turkish [23], Chinese [28], Pakistani [31], Polish [30], Indian [29], and Italian [20]. All of the studies investigated the canine, the first and second premolar, and the second molar, with the exception of one study [29], in which only the second molar was included. All of the studies enrolled both male and female subjects, and the sample sizes ranged from 300 [29] to 718 [30]; the male-to-female ratio was equal [23] or similar [28, 29] to 1:1, or in favour of females [20, 30, 31]. The age ranges of the investigated subjects among the studies were similar, with an overall range between 6 and 18 years. All of the subjects included in these studies had to be healthy, with no major nutritional, metabolic, or growth impairment.

**Figure 3:**
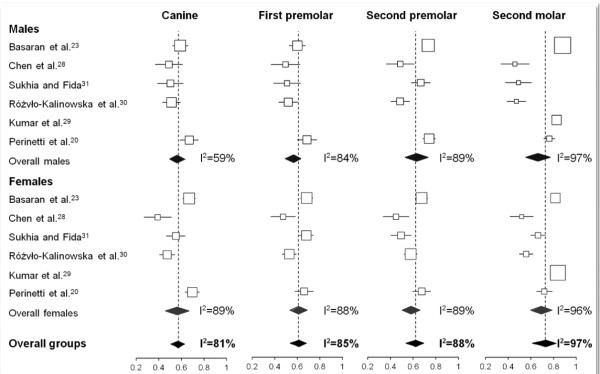
**Forest plots for the correlation coefficients between the dental maturation and the CVM stages.** According to the four mandibular teeth and sexes. Blocks, point estimates; lines, 95% confidence intervals.

All of the studies saw a positive correlation between dental stage and cervical vertebral maturation, which ranged from moderate to high values (Table 1 and Figure 3). The majority of the studies showed differential behaviour of the teeth between the sexes [23, 28, 30, 31], and one study [20] reported little differences between the sexes and the teeth, while another study [29] demonstrated similarities between males and females only when the second molar was investigated.

For the diagnostic indications of the specific dental maturational stages in the identification of skeletal maturational stages, two studies [23, 30] did not provide information; the study [28] on Chinese subjects suggested that stage G of the canine for males and stage F of the second molar for females might signify the beginning of the pubertal growth spurt; one study reported that the onset of the pubertal growth phase (i.e. CS3) might be diagnosed by stage H of the first premolar in males and stage G of the second molar in females. A further study [29] on only the second molar reported that for both male and female subjects, maturation up to stage E is indicative of a pre-pubertal growth phase (i.e. CS2), stages F and G are indicative of the pubertal growth phase (i.e. CS3 and CS4, respectively), and stage H is mainly present during a post-pubertal growth phase. The last study [20] reported that the canine up to stage F and the first and second premolar and second molar up to stage E are indicative of a pre-pubertal growth phase (CS1 and CS2, with no distinctions) and that the second molar at stage H is mainly present during the post-pubertal growth phase. While the conclusions from three studies [28, 29, 31] were only based on the percentage distributions of the different maturational stages across the skeletal maturational stages, those from the last study [20] were based on a diagnostic performance analysis. Finally, regarding the diagnostic usefulness of the dental maturational staging in the identification of skeletal maturity, the studies suggested reliable use for the diagnosis of all of the skeletal maturational stages [23, 29, 31] or for the onset of the pubertal growth spurt [28]. One study [30] reported that dental maturity might be useful only as an initial assessment of the growth phase. The last study [20] suggested a reliable diagnostic use of dental maturation only for the identification of the pre-pubertal growth phase.

#### Quality analysis and risk of bias in individual studies

The results of the quality analysis are given in Table 2. The quality was high in only one study [20], medium in three studies [28, 29, 31], and low in the remaining two studies [23, 30].

The sample description was classified as adequate in all of the studies, with clear indications of the inclusion/exclusion criteria. Also, the data regarding the age and sex distributions were satisfactory in all of the studies.

**Table 2:** **Quality evaluation of the six studies included in the meta-analysis**

Study	Sample description	Method error analysis	Adequacy of statistics	Previous estimate of sample size	Blinding in measurements	Quality score	Judged quality standard
Başaran et al. [23]	Full	No	Partial	No	No	3	Low
Chen et al. [28]	Full	Full	Partial	No	No	5	Medium
Sukhia and Fida [31]	Full	Full	Partial	No	No	5	Medium
Różyło-Kalinowska et al. [30]	Full	No	Partial	No	No	3	Low
Perinetti et al. [20]	Full	Full	Full	No	Yes	7	High
Kumar et al. [29]	Full	Full	Partial	No	No	5	Medium

Four studies [20, 28, 29, 31] included a full method error analysis, for both the dental and cervical maturational staging, based on inter-operator or intra-operator test-retest recordings. One study [23] reported that a method error analysis was performed, but no data were shown; thus, the point was not assigned. The procedures used to assess method error were kappa statistics [20, 28, 29] or the Bland-Altman analysis [31]. In all of these four studies [20, 28, 29, 31], satisfactory levels of intra-operator and inter-operator agreement were reached.

Adequacy of statistics was judged as full in only one study [20] and partial in all of the rest [23, 28–31]. Although the use of parametric/non-parametric methods and the other tests used were appropriate in all of the studies, five of these investigations [23, 28–31] lacked correct analysis of the diagnostic performance, which limited the statistical analysis to percentage distributions of the dental maturational stages across CS1 to CS6, and to the correlation coefficients between the two staging systems.

Prior estimate of sample size was not performed in any of the studies, although the sample sizes can be considered large, as they were composed of several hundreds of subjects. Finally, blinding in measurements (for both the dental and cervical vertebral maturational staging) was reported in only one study [20].

#### Distributions of the dental maturational stages and growth phases, and the degrees of correlation

The distributions of the dental maturational stages among the different growth phases for each investigated tooth are given in Tables 3, 4, 5, and 6. All of the investigated teeth showed a spread diffusion of their maturational stages across the different growth phases. This behaviour is seen both for the six studies included and for the corresponding overall distributions. Regarding these overall data, the greatest distributions of a given tooth and maturational stage were seen for the canine and the first and second premolar, all at stage H at the pubertal growth phase, with values of 24.2%, 22.7%, and 15.1%, respectively, and for the second molar stage G at the pubertal growth phase at 19.6%.

The degrees of correlation between the dental and skeletal maturational stages according to the sexes for each investigated tooth are shown in Figure 3. For each investigated tooth, all of the overall correlation coefficients were statistically significant (*p* < 0.001, at least). In particular, when pooling the male and female subjects, the overall correlation coefficients for the canine, the first and second premolar, and the second molar were 0.57, 0.62, 0.62, and 0.73, respectively. The heterogeneity among the studies was generally proportional to the correlation coefficients, with *I*^2^ values from 81% to 97% for the canine and second molar, respectively. This heterogeneity was also similar between the sexes, with the exception for the canine, in which a greater *I*^2^ of 89% was seen for female subjects as compared to that of 59% seen for male subjects.

#### Diagnostic performances

A total of 227 positive LHRs were calculated. Only 19 (8.4%) of these positive LHRs were ≥10.0, with 18 related to the pre-pubertal growth phase (all of the teeth) and only 1 related to the post-pubertal growth phase (the second molar). Moreover, a total of 48 overall positive LHRs were obtained in the meta-analysis. Only 4 (8.3%) of these overall positive LHRs were ≥10.0, with all related to the pre-pubertal growth phase (canine, stages E and G; first premolar, stage E; and second molar, stage D). For the pubertal growth phase, the positive LHRs retrieved in each study were generally below 2.0, with the greatest value of 9.1 seen for the second molar stage G in one study [29]. However, the greatest overall positive LHR for the identification of this growth phase was 2.3, seen for the second molar stage G. Similarly, for the post-pubertal growth phase, the positive LHRs retrieved in each study were generally below 3.0, with the exceptions of stage H of the second premolar and second molar, which were greater, although in only one study [29] was the threshold reached, with a value of 206.4, while in another study [20], a maximum value of 9.1 was seen (both second molar, stage H). The greatest overall positive LHRs for the identification of this growth phase was 6.7, as seen for the second molar stage H.

### Discussion

The present meta-analysis has reappraised the diagnostic performances of the maturation stages of four mandibular teeth for the identification of the circumpubertal growth phase in individual subjects. The data show that in spite of the high correlation coefficients seen, according to which dental maturation has been proposed as a reliable indicator of skeletal maturity (Table 1), the diagnostic performance of these dental maturational stages is limited for each of the investigated teeth. Moreover, the repeatability of the CVM method that has been reported low for untrained operators [32] would not constitute a limitation herein since in most of the included studies [20, 28, 29, 31], a satisfactorily repeatability for the CVM staging has been shown.

**Table 3:** **Percentage distributions of the maturation stages of the mandibular canine among different growth phases**

Growth phase	Dental stage	Başaran et al.[23]	Chen et al.[28]	Sukhia and Fida[31]	Różyło-Kalinowska et al.[30]	Perinetti et al.[20]	Overall
Pre-pubertal	D	1.7%	0	0	0	1.1%	0.6% (0.2-1.8)
	E	7.5%	0.3%	2.6%	0.8%	8.5%	2.9% (1.2-6.5)
	F	15.1%	11.6%	20.3%	4.6%	24.0%	15.5% (14.0-17.2)
	G	6.6%	16.2%	11.6%	18.3%	15.0%	13.0% (9.2-18.0)
	H	1.2%	11.6%	12.1%	10.6%	10.2%	8.1% (5.2-12.4)
Pubertal	D	0	0	0	0	0	0.1% (0.0-0.4)
	E	0	0	0.3%	0.1%	0	0.2% (0.1-0.5)
	F	1.2%	2.0%	3.7%	0.8%	1.1%	1.6% (0.9-2.9)
	G	3.4%	16.9%	9.7%	11.4%	5.4%	8.4% (5.1-13.5)
	H	25.6%	24.5%	33.7%	24.5%%	14.4%	24.2% (19.3-29.9)
Post-pubertal	D	0	0	0	0	0	0.1% (0.0-0.4)
	E	0	0	0	0	0	0.1% (0.0-0.4)
	F	0	0.3%	0	0	0	0.2% (0.0-0.5)
	G	0.5%	2.3%	0.3%	1.7%	1.1%	1.2% (0.7-2.2)
	H	36.9%	14.2%	5.8%	26.9%	19.2%	18.4% (11.1-29.1)

Percentage distributions were computed for the whole sample within each study. Overall percentage distributions are shown as means (95% CI).

Only studies that scored dental maturation according to the method described by Demirjian et al [15] were included here, as this method consists of distinct details based on shape criteria and proportion of root length, using relative values to the crown height rather than absolute lengths. Foreshortened or elongated projections of developing teeth will not affect the reliability of this assessment [15]. On the other hand, because of the different staging of the hand-and-wrist [9, 11] and cervical vertebral [3] maturational methods, only the latter was considered here as the indicator of the growth phase. In particular, the CVM method has been shown to be of reliable and simple application, making this assessment widely used nowadays both in research and in clinical practice. Nonetheless, the CVM method requires a lateral head film, which might be available as a pre-treatment record, but should not be obtained later only for the purpose of monitoring the growth phase, as in this case the optimal treatment timing would be delayed until after the diagnosis. As a disadvantage, the hand-and-wrist maturation method requires additional X-ray exposure. Therefore, from a research and clinical standpoint, dental maturation was proposed a long time ago as a further useful method for assessing the growth phase in individual subjects [12].

**Table 4:** **Percentage distributions of the maturation stages of the mandibular first premolar among different growth phases**

Growth phase	Dental stage	Başaran et al.[23]	Chen et al.[28]	Sukhia and Fida[31]	Różyło-Kalinowska et al.[30]	Perinetti et al.[20]	Overall
Pre-pubertal	D	4.2%	0	0.3%	0.1%	1.7%	0.8% (0.2-2.8)
	E	8.5%	1.0%	7.9%	2.1%	15.8%	5.4% (2.6-10.8)
	F	11.2%	13.2%	15.8%	8.4%	18.9%	13.1% (9.7-17.3)
	G	5.8%	17.5%	13.9%	13.0%	14.1%	13.1% (9.7-17.3)
	H	0.7%	7.9%	8.7%	10.7%	8.2%	6.5% (4.1-10.3)
Pubertal	D	0	0	0	0	0	6.5% (4.1-10.3)
	E	0.2%	0.3%	0.8%	0.1%	0.3%	6.5% (4.1-10.3)
	F	1.0%	4.6%	4.7%	3.1%	2.0%	5.5% (2.6-11.2)
	G	4.1%	18.9%	8.2%	10.2%	4.8%	8.1% (4.7-13.7)
	H	24.9%	19.5%	33.7%	23.6%	13.8%	22.7% (17.5-28.8)
Post-pubertal	D	0	0	0	0	0	6.5% (4.1-10.3)
	E	0	0	0	0	0	6.5% (4.1-10.3)
	F	0	0.3%	0.3%	0.3%	0.3%	0.3% (0.1-0.6)
	G	0.7%	3.6%	5.0%	2.0%	1.7%	2.3% (1.2-4.2)
	H	36.8%	12.9%	0.8%	26.4%	18.4%	15.6% (9.1-25.4)

Percentage distributions were computed for the whole sample within each study. Overall percentage distributions are shown as means (95% CI).

**Table 5:** **Percentage distributions of the maturation stages of the mandibular second premolar among different growth phases**

Growth phase	Dental stage	Başaran et al.[23]	Chen et al.[28]	Sukhia and Fida[31]	Różyło-Kalinowska et al.[30]	Perinetti et al.[20]	Overall
Pre-pubertal	D	8.8%	0	3.4%	1.8%	9.0%	4.0% (1.9-8.2)
	E	12.7%	1.7%	10.3%	2.9%	20.3%	7.3% (3.6-14.3)
	F	7.6%	18.9%	16.6%	11.6%	15.8%	13.5% (10.0-18.1)
	G	1.5%	14.2%	11.3%	14.2%	9.6%	8.8% (5.5-14.0)
	H	0.5%	5.0%	5.0%	4.3%	3.4%	3.5% (2.2-5.5)
Pubertal	D	0.0	0	0	0	0	0.1% (0.0-0.4)
	E	1.4%	0.3%	2.6%	0.6%	1.1%	1.2% (0.6-2.2)
	F	2.9%	8.9%	8.4%	7.3%	2.5%	5.6% (3.5-8.6)
	G	6.4%	22.2%	11.6%	14.6%	9.0%	11.9% (7.9-17.6)
	H	19.5%	11.9%	24.7%	14.5%	8.2%	15.1% (10.8-20.8)
Post-pubertal	D	0	0	0	0	0	0.1% (0.0-0.4)
	E	0	0	0	0	0	0.1% (0.0-0.4)
	F	0	1.7%	0.3%	1.0%	0.8%	0.9% (0.4-1.7)
	G	0.8%	4.0%	0.5%	5.7%	6.2%	2.9% (1.4-5.5)
	H	36.6%	11.3%	5.3%	21.9%	13.3%	15.3% (8.2-26.7)

Percentage distributions were computed for the whole sample within each study. Overall percentage distributions are shown as means (95% CI).

The main limitations of the studies included that were judged to be of low and medium quality were the lack of a full analysis of the diagnostic performance and of blinding for the measurements [23, 28–31]. Moreover, the two studies [23, 30] with low quality also lacked an internal method error analysis. However, in spite of these limitations, the very similar protocols of these cross-sectional studies render them highly comparable.

**Table 6:** **Percentage distributions of the maturation stages of the mandibular second molar among different growth phases**

Growth phase	Dental Stage	Başaran et al.[23]	Chen et al.[28]	Sukhia and Fida[31]	Różyło-Kalinowska et al.[30]	Kumar et al.[29]	Perinetti et al.[20]	Overall
Pre-pubertal	D	11.7%	1.0%	4.5%	2.4%	5.7%	18.4%	5.4% (2.6-10.8)
	E	9.8%	6.3%	11.1%	4.6%	19.7%	21.8%	10.8% (6.6-17.3)
	F	5.8%	19.5%	14.5%	8.8%	5.7%	10.5%	10.0% (6.7-14.6)
	G	0.5%	11.9%	15.3%	19.0%	0.7%	7.9%	6.7% (3.7-11.9)
	H	0	1.0%	1.3%	0.3%	0	0.3%	0.5% (0.2-1.2)
Pubertal	D	0.5%	0.3%	0	0	0	0.6%	0.4% (0.2-0.7)
	E	2.5%	1.3%	2.9%	1.1%	0.7%	1.4%	1.7% (1.1-2.6)
	F	4.2%	13.9%	5.0%	4.3%	21.7%	4.2%	7.2% (3.6-13.8)
	G	12.7%	23.2%	25.8%	28.0%	22.0%	10.5%	19.6% (14.3-26.2)
	H	10.2%	4.6%	13.7%	3.3%	0.3%	4.2%	5.3% (2.9-9.4)
Post-pubertal	D	0	0	0	0	0	0	0.1% (0.0-0.4)
	E	0	0.3%	0	0	0	0	0.2% (0.1-0.4)
	F	0	2.0%	0.3%	0.6%	0	1.7%	0.7% (0.3-1.7)
	G	9.0%	7.9%	1.8%	17.0%	2.3%	8.2%	6.5% (3.7-11.2)
	H	28.5%	6.6%	3.9%	11.0%	21.3%	10.5%	11.6% (6.5-19.9)

Percentage distributions were computed for the whole sample within each study. Overall percentage distributions are shown as means (95% CI).

**Table 7:** **Positive LHRs for the maturation stages of the mandibular canine for diagnosis of different growth phases**

Growth phase	Dental stage	Başaran et al.[23]	Chen et al.[28]	Sukhia and Fida[31]	Różyło-Kalinowska et al.[30]	Perinetti et al.[20]	Overall^a^
Pre-pubertal	D	–	–	–	–	–	–
	E	–	–	*11.5* (1.5-88.7)	*11.4* (1.4-94.3)	–	*11.4* (2.6-49.8)
	F	*26.6* (12.5-56.2)	7.6 (3.5-16.5)	6.3 (3.7-10.7)	*10.5* (4.4-24.6)	*14.9* (5.6-39.8)	*11.1* (6.4-19.2)
	G	3.5 (2.2-5.8)	1.3 (0.9-1.7)	1.3 (0.9-1.9)	2.7 (2.1-3.3)	1.6 (1.0-2.5)	1.9 (1.3-2.8)
	H	0.0 (0.0-0.1)	0.5 (0.3-0.6)	0.4 (0.3-0.5)	0.4 (0.3-0.5)	0.2 (0.2-0.3)	0.4 (0.3-0.4)
Pubertal	D	–	–	–	–	–	–
	E	–	–	0.1 (0.0-0.9)	0.3 (0.0-2.3)	–	0.2 (0.2-0.3)
	F	0.2 (0.1-0.4)	0.2 (0.1-0.5)	0.2 (0.1-0.3)	0.3 (0.1-0.7)	0.2 (0.1-0.5)	0.2 (0.2-0.3)
	G	1.1 (0.7-1.8)	1.2 (0.9-1.6)	0.9 (0.6-1.3)	1.0 (0.8-1.2)	1.3 (0.8-2.0)	1.1 (0.9-1.2)
	H	1.6 (1.4-1.7)	1.2 (1.0-1.5)	2.1 (1.7-2.6)	1.1 (1.0-1.3)	1.9 (1.5-2.3)	0.9 (0.5-1.5)
Post-pubertal	D	–	–	–	–	–	–
	E	–	0.2 (0.0-1.5)	–	–	–	–
	F	–	0.1 (0.0-0.9)	–	–	–	–
	G	0.1 (0.0-0.3)	0.3 (0.2-0.7)	0.2 (0.0-1.3)	0.1 (0.1-0.2)	0.2 (0.1-0.6)	0.2 (0.1-0.3)
	H	2.3 (2.0-2.6)	1.9 (1.6-2.3)	2.0 (1.7-2.3)	1.9 (1.7-2.1)	3.1 (2.5-3.7)	2.2 (1.9-2.5)

Data are presented as means (95% confidence interval). ^a^Null values and values equal to zero not included. The symbol ‘**--**’ represents null value indicating that no cases for the given maturational stage coincided with the corresponding growth phase. Values in italics denote an overall positive LHR of 10 or more.

On the basis of the distribution of the different dental maturational stages across the CVM stages and their corresponding correlation coefficients, five [23, 28–31] of the studies included indicated the diagnostic usefulness of dental maturity in assessing the circumpubertal growth phases (Table 1). However, two [23, 30] of these five studies did not provide any diagnostic indications on the specific dental stage in the identification of the skeletal maturation phase. Moreover, where indications were given, different results are seen among these studies. For the identification of the pubertal growth phase, i.e. CS3 and CS4, the canine, first premolar, and second molar were all suggested to have diagnostic usefulness, with some differences among the sexes and stages (Table 1).

**Table 8:** **Positive LHRs for maturation stages of the mandibular first premolar for diagnosis of different growth phases**

Growth phase	Dental stage	Başaran et al.[23]	Chen et al.[28]	Sukhia and Fida[31]	Różyło-Kalinowska et al.[30]	Perinetti et al.[20]	Overall^a^
Pre-pubertal	D	–	–	–	–	–	–
	E	*104.5* (14.5-750.4)	4.6 (0.5-43.2)	*11.5* (3.6-36.9)	*28.5* (3.8-214.8)	*39.3* (5.5-280.8)	*21.5* (8.1-56.7)
	F	*23.0* (10.1-52.1)	4.0 (2.3-7.0)	3.6 (2.3-5.8)	4.8 (3.0-7.4)	5.9 (2.9-11.9)	5.8 (3.5-9.7)
	G	2.5 (1.6-4.1)	1.2 (0.9-1.6)	1.2 (0.1-1.7)	2.0 (1.6-2.6)	1.5 (1.0-2.4)	1.6 (1.2- 2.2)
	H	0.0 (0.0-0.1)	0.4 (0.3-0.5)	0.3 (0.2-0.4)	0.4 (0.3-0.5)	0.2 (0.13-0.3)	0.2 (0.1-0.4)
Pubertal	D	–	–	–	–	–	–
	E	0.0 (0.0-0.3)	0.4 (0.0-4.1)	0.1 (0.0-0.4)	0.1 (0.0-0.9)	0.1 (0.0-0.5)	0.1 (0.0-0.2)
	F	0.2 (0.1-0.5)	0.4 (0.3-0.8)	0.3 (0.2-0.5)	0.6 (0.4-1.0)	0.4 (0.2-0.8)	0.4 (0.3-0.6)
	G	1.5 (0.9-2.4)	1.2 (0.9-1.5)	0.5 (0.3-0.7)	1.2 (0.9-1.5)	1.1 (0.7-1.9)	1.0 (0.9-1.2)
	H	1.5 (1.4-1.7)	1.2 (0.9-1.6)	4.0 (2.9-5.4)	1.1 (1.0-1.2)	2.0 (1.6-2.5)	0.9 (0.5-1.8)
Post-pubertal	D	–	–	–	–	–	–
	E	–	0.2 (0.0-1.5)	–	–	–	0.2 (0.0-1.5)
	F	–	0.1 (0.0-0.6)	0.2 (0.0-1.4)	0.1 (0.0-0.2)	0.1 (0.0-0.4)	0.1 (0.0-0.2)
	G	0.1 (0.0-0.3)	0.5 (0.3-0.8)	3.5 (2.7-4.6)	0.2 (0.1-0.4)	0.4 (0.2-0.8)	0.4 (0.1-1.9)
	H	2.4 (2.1-2.7)	2.3 (1.8-2.9)	0.3 (0.1-0.8)	1.9 (1.7-2.1)	3.3 (2.7-4.0)	2.2 (1.7-2.8)

Data are presented as means (95% CI). ^a^Null values and values equal to zero not included. The symbol ‘**--**’ represents null value indicating that no cases for the given maturational stage coincided with the corresponding growth phase. Values in italics denote an overall positive LHR of 10 or more.

**Table 9:** **Positive LHRs for maturation stages of the mandibular second premolar for diagnosis of different growth phases**

Growth phase	Dental stage	Başaran et al.[23]	Chen et al.[28]	Sukhia and Fida[31]	Różyło-Kalinowska et al.[30]	Perinetti et al.[20]	Overall^a^
Pre-pubertal	D	–	–	–	–	–	–
	E	*19.6* (9.6-39.8)	7.6 (0.9-64.1)	4.5 (2.3-8.7)	*10.0* (3.5-28.8)	*12.8* (4.8-34.1)	9.8 (5.1-19.0)
	F	5.5 (3.3-9.4)	2.7 (1.9-3.9)	2.2 (1.5-3.2)	2.7 (2.0-3.6)	3.3 (1.8-5.9)	2.9 (2.3-3.8)
	G	0.4 (0.2-0.9)	0.8 (0.6-1.1)	1.1 (0.7-1.5)	1.3 (1.1-1.6)	0.4 (0.3-0.6)	0.8 (0.5-1.2)
	H	0.0 (0.0-0.1)	0.3 (0.2-0.5)	0.2 (0.1-0.3)	0.2 (0.2-0.3)	0.1 (0.1-0.2)	0.1 (0.1-0.3)
Pubertal	D	–	–	–	–	–	–
	E	0.2 (0.1-0.5)	0.3 (0.0-2.2)	0.3 (0.1-0.6)	0.3 (0.1-0.9)	0.2 (0.1-0.6)	0.3 (0.2-0.4)
	F	0.9 (0.5-1.5)	0.6 (0.4-0.8)	0.6 (0.4-0.8)	1.0 (0.7-1.3)	0.6 (0.3-1.1)	0.7 (0.5-0.9)
	G	6.3 (3.5-11.3)	1.6 (1.2-2.1)	1.1 (0.8-1.6)	1.3 (1.0-1.5)	2.1 (1.5-3.0)	1.8 (1.2-2.8)
	H	1.2 (1.1-1.4)	1.0 (0.7-1.4)	2.7 (2.0-3.7)	0.9 (0.8-1.1)	1.8 (1.3-2.7)	0.8 (0.4-1.6)
Post-pubertal	D	–	–	–	–	–	–
	E	–	0.2 (0.0-1.5)	–	–	–	0.2 (0.0-1.5)
	F	–	0.3 (0.1-0.7)	0.2 (0.0-1.1)	0.1 (0.1-0.3)	0.2 (0.1-0.6)	0.2 (0.1-0.3)
	G	0.2 (0.1-0.4)	0.5 (0.3-0.9)	0.4 (0.1-1.4)	0.5 (0.4-0.7)	1.3 (0.9-1.9)	0.5 (0.3-0.9)
	H	3.1 (2.6-3.6)	3.3 (2.4-4.5)	2.7 (2.2-3.4)	2.9 (2.5-3.4)	4.5 (3.2-6.2)	3.1 (2.7-3.5)

Data are presented as means (95% CI). ^a^Null values and values equal to zero not included. The symbol ‘**--**’ represent null value indicating that no cases for the given maturational stage coincided with the corresponding growth phase. Values in italics denote an overall positive LHR of 10 or more.

The percentage distributions of the dental maturational stages across the different growth phases (Tables 3, 4, 5, and 6) were computed for the whole sample within each study, instead of within each growth phase, as previously reported, as this better resembles the diagnostic capabilities of the dental maturational stages in the identification of the different growth phases. According to this part of the meta-analysis, noteworthy differences in the distributions among the studies were seen (*I*^2^ generally above 50%, not shown). This shows that the dental formation follows differential timing with regard to the growth phases [5, 9, 10] among the different ethnic populations, rendering unique reference scores inapplicable. Also, the correlation coefficients between the dental and skeletal maturational stages varied significantly among the six studies, although they were statistically significant in all of the cases (Figure 3). The overall coefficients (with pooling of the male and female subjects) varied from 0.57 for the canine to 0.73 for the second molar. However, the heterogeneity among the studies was proportional to these correlation coefficients, thus showing that the greatest correlation coefficients were also associated with the greatest variability, as was seen for the second molar (overall, *I*^2^ up to 97%). In terms of the correlation coefficients, differences were seen between the sexes within the same tooth and study.

As the main goal of the present meta-analysis, an appraisal of the diagnostic performances of the dental maturational stages in the identification of the growth phase was performed by calculation of the positive LHRs from the previously reported data. According to this appraisal, in only a few cases were positive LHRs retrieved in each of the studies that were above the required threshold for satisfactory performance (Tables 7, 8, 9, and 10). In particular, some of the maturational stages of the canine, first premolar, and second molar were seen to be associated mostly with the pre-pubertal growth phase. The maturational stages did not reach a satisfactorily level of diagnostic performance for any of the investigated teeth in the identification of the pubertal and post-pubertal growth phases. The only exception was for the second molar (stage H), which yielded a positive LHR of 206.4 for the identification of the post-pubertal growth phase in one study [29]. All of this evidence would thus not support the conclusions of most of the studies included [23, 28, 29, 31] (Table 1), in which clear indications for a given maturational stage in the identification of the growth phase were reported. However, according to the overall positive LHRs, very little diagnostic performances were uncovered (Tables 7, 8, 9, and 10). Of interest, the present appraisal considered three growth phases by clustering the six CVM stages; the merging of which allowed the retrieval of higher positive LHRs than those obtained by using the six skeletal maturational stages separately. Therefore, future studies that suggest the use of an indicator for a given condition should have conclusions based on a correct and full diagnostic performance analysis, as the strength of correlations between two scales is not sufficient to assess diagnostic capabilities.

**Table 10:** **Positive LHRs for maturation stages of the mandibular second molar for diagnosis of different growth phases**

Growth phase	Dental stage	Başaran et al.[23]	Chen et al.[28]	Sukhia and Fida[31]	Różyło-Kalinowska et al.[30]	Kumar et al.[29]	Perinetti et al.[20]	Overall^a^
Pre-pubertal	D	*48.0* (15.3-150.7)	4.6 (0.5-43.2)	–	*22.8* (3.0-174.6)	–	*22.8* (5.7-91.7)	*25.2* (10.9-58.4)
	E	8.1 (4.7-13.9)	5.8 (2.2-15.0)	4.4 (2.3-8.2)	7.8 (3.7-16.7)	*63.7* (15.9-255.1)	*10.8* (4.5-26.0)	8.6 (5.1-14.5)
	F	2.8 (1.7-4.6)	1.9 (1.4-2.5)	3.2 (2.0-5.0)	3.4 (2.3-5.0)	0.6 (0.6-0.9)	1.2 (0.8-2.0)	1.9 (1.1-3.1)
	G	0.0 (0.0-0.2)	0.6 (0.4-0.8)	0.6 (0.5-0.8)	0.8 (0.7-0.9)	0.1 (0.0-0.2)	0.3 (0.2-0.4)	0.4 (0.2-0.6)
	H	–	0.1 (0.0-0.4)	0.1 (0.0-0.2)	0.0 (0.0-0.2)	–	0.0 (0.0-0.1)	0.1 (0.0-0.1)
Pubertal	D	0.1 (0.0-0.3)	0.4 (0.0-4.1)	–	0.1 (0.0-1.1)	–	0.1 (0.0-0.5)	0.1 (0.1-0.3)
	E	0.6 (0.3-1.0)	0.3 (0.1-0.7)	0.3 (0.2-0.5)	0.4 (0.2-0.9)	0.0 (0.0-0.2)	0.2 (0.1-0.6)	0.3 (0.2-0.5)
	F	1.7 (1.0-2.8)	0.8 (0.6-1.2)	0.4 (0.2-0.6)	0.8 (0.5-1.2)	4.7 (2.9-7.7)	1.3 (0.8-2.2)	1.2 (0.6-2.2)
	G	3.1 (2.3-4.2)	1.5 (1.2-2.0)	1.7 (1.3-2.1)	1.3 (1.2-1.5)	9.1 (4.7-17.5)	2.5 (1.8-3.4)	2.3 (1.6-3.3)
	H	0.8 (0.7-1.0)	0.8 (0.4-1.5)	2.9 (1.8-4.6)	0.5 (0.3-0.8)	0.0 (0.0-0.1)	1.5 (0.9-2.6)	0.9 (0.4-2.0)
Post-pubertal	D	–	–	–	–	–	–	–
	E	–	0.2 (0.0-1.5)	–	–	–	–	0.2 (0.0-1.5)
	F	–	0.3 (0.1-0.6)	0.2 (0.0-1.4)	0.1 (0.0-0.3)	–	0.5 (0.2-1.0)	0.3 (0.1-0.5)
	G	1.1 (0.8-1.5)	1.1 (0.8-1.5)	0.7 (0.4-1.3)	0.9 (0.8-1.0)	0.3 (0.2-0.7)	1.7 (1.2-2.5)	1.0 (0.7-1.1)
	H	4.7 (3.7-6.0)	5.8 (3.3-10.3)	4.1 (2.8-6.0)	7.6 (5.0-11.5)	*206.4* (29.2-1,461.4)	9.1 (5.4-15.3)	6.7 (4.4-10.1)

Data are presented as means (95% CI). ^a^Null values and values equal to zero not included. The symbol ‘**--**’ represents null value indicating that no cases for the given maturational stage coincided with the corresponding growth phase. Values in italics denote an overall positive LHR of 10 or more.

Finally, even though the present meta-analysis did not include the radiographic hand-and-wrist maturational method, the CVM method *per se* can be considered as a reliable indicator of skeletal maturation; moreover, the great agreement between the results obtained from the studies included (especially in terms of the positive LHRs) makes the present conclusions reliable.

#### Clinical implications

In consideration of the diagnostic performance analysis presented here, dental maturity is not a reliable indicator of the growth phase in individual subjects. The present meta-analysis has thus revealed that the conclusions reported in previous studies were not actually supported by the results obtained in those studies [23, 28–31]. In this regard, a further investigation [33] using a subset of a study [20] included in the present meta-analysis has revealed that even the maturational combination of the mandibular canine and second molar would have no diagnostic potential in the identification of the pubertal growth phase. Therefore, whenever available, hand-and-wrist [9, 11] or cervical vertebral [3, 13] maturational methods remain preferable for the determination of the growth phase, and hence of treatment timing, in individual growing subjects. Few exceptions were seen for the canine up to stage F, the first premolar up to stage E, and the second molar up to stage D, which might be satisfactorily used for diagnosis of the pre-pubertal growth phase. However, considering that the diagnostic accuracy of the early mixed and intermediate mixed dentition for the identification of the pre-pubertal growth phase has been demonstrated [5, 9, 10], dental emergence can be used instead of dental maturation, thus avoiding the need for an X-ray, at least for the identification of the pre-pubertal growth phase. Moreover, the diagnosis of a pre-pubertal growth phase by dental maturity, or even by dental emergence, does not provide precise information on the duration of this growth phase up to the beginning of the subsequent pubertal growth spurt. Finally, the invasiveness of radiographical indicators has to be taken into account in clinical practice, at least until non-invasive biomarkers [8, 14, 34] of growth phase will be available for routine activities.

## Conclusions

The present meta-analysis has the following conclusions:

1. Dental maturity and skeletal maturity are significantly correlated, although there are differences across ethnic populations.
2. In spite of these correlations, the diagnostic performance of dental maturity for the identification of growth phases, and especially of the pubertal growth spurt, is very limited.
3. The determination of dental maturity for the assessment of treatment timing for skeletal malocclusion is not recommended.

## Electronic supplementary material

Additional file 1: **PRISMA checklist.** A document showing the PRISMA checklist. (DOC 67 KB)

## References

[1] Perinetti G, Cordella C, Pellegrini F, Esposito P (2008). The prevalence of malocclusal traits and their correlations in mixed dentition children: results from the Italian OHSAR Survey. Oral Health Prev Dent.

[2] Proffit WR, Fields HW, Moray LJ (1998). Prevalence of malocclusion and orthodontic treatment need in the United States: estimates from the NHANES III survey. Int J Adult Orthodon Orthognath Surg.

[3] Baccetti T, Franchi L, McNamara JAJ (2005). The cervical vertebral maturation (CVM) method for the assessment of optimal treatment timing in dentofacial orthopedics. Semin Orthod.

[4] Hagg U, Pancherz H (1988). Dentofacial orthopaedics in relation to chronological age, growth period and skeletal development. An analysis of 72 male patients with Class II division 1 malocclusion treated with the Herbst appliance. Eur J Orthod.

[5] Bjork A, Helm S (1967). Prediction of the age of maximum puberal growth in body height. Angle Orthod.

[6] Baccetti T, Franchi L, De Toffol L, Ghiozzi B, Cozza P (2006). The diagnostic performance of chronologic age in the assessment of skeletal maturity. Prog Orthod.

[7] Melsen B (1975). Palatal growth studied on human autopsy material. A histologic microradiographic study. Am J Orthod.

[8] Perinetti G, Baccetti T, Di Leonardo B, Di Lenarda R, Contardo L (2011). Dentition phase and chronological age in relation to gingival crevicular fluid alkaline phosphatase activity in growing subjects. Prog Orthod.

[9] Hagg U, Taranger J (1982). Maturation indicators and the pubertal growth spurt. Am J Orthod.

[10] Franchi L, Baccetti T, De Toffol L, Polimeni A, Cozza P (2008). Phases of the dentition for the assessment of skeletal maturity: a diagnostic performance study. Am J Orthod Dentofacial Orthop.

[11] Greulich WW, Pyle SI (1959). Radiographic atlas of skeletal development of the hand and wrist.

[12] Chertkow S (1980). Tooth mineralization as an indicator of the pubertal growth spurt. Am J Orthod.

[13] Hassel B, Farman AG (1995). Skeletal maturation evaluation using cervical vertebrae. Am J Orthod Dentofacial Orthop.

[14] Perinetti G, Baccetti T, Contardo L, Di Lenarda R (2011). Gingival crevicular fluid alkaline phosphatase activity as a non-invasive biomarker of skeletal maturation. Orthod Craniofac Res.

[15] Demirjian A, Goldstein H, Tanner JM (1973). A new system of dental age assessment. Hum Biol.

[16] Coutinho S, Buschang PH, Miranda F (1993). Relationships between mandibular canine calcification stages and skeletal maturity. Am J Orthod Dentofacial Orthop.

[17] Krailassiri S, Anuwongnukroh N, Dechkunakorn S (2002). Relationships between dental calcification stages and skeletal maturity indicators in Thai individuals. Angle Orthod.

[18] Deeks JJ, Altman DG (2004). Diagnostic tests 4: likelihood ratios. BMJ.

[19] Greenhalgh T (1997). How to read a paper. Papers that report diagnostic or screening tests. BMJ.

[20] Perinetti G, Contardo L, Gabrieli P, Baccetti T, Di Lenarda R (2012). Diagnostic performance of dental maturity for identification of skeletal maturation phase. Eur J Orthod.

[21] Liberati A, Altman DG, Tetzlaff J, Mulrow C, Gotzsche PC, Ioannidis JP, Clark M, Devereaux PJ, Kleijnen J, Moher D (2009). The PRISMA statement for reporting systematic reviews and meta-analyses of studies that evaluate health care interventions: explanation and elaboration. J Clin Epidemiol.

[22] Landis JR, Koch GG (1977). The measurement of observer agreement for categorical data. Biometrics.

[23] Basaran G, Ozer T, Hamamci N (2007). Cervical vertebral and dental maturity in Turkish subjects. Am J Orthod Dentofacial Orthop.

[24] Perinetti G, Contardo L (2009). Posturography as a diagnostic aid in dentistry: a systematic review. J Oral Rehabil.

[25] Perinetti G, Contardo L, Primozic J, Di Lenarda R (2011). Associations between the masticatory system and muscle activity of other body districts. A meta-analysis of surface electromyography studies. J Electromyogr Kinesiol.

[26] Lau J, Ioannidis JP, Schmid CH (1997). Quantitative synthesis in systematic reviews. Ann Intern Med.

[27] Ried K (2006). Interpreting and understanding meta-analysis graphs–a practical guide. Aust Fam Physician.

[28] Chen J, Hu H, Guo J, Liu Z, Liu R, Li F, Zou S (2010). Correlation between dental maturity and cervical vertebral maturity. Oral Surg Oral Med Oral Pathol Oral Radiol Endod.

[29] Kumar S, Singla A, Sharma R, Virdi MS, Anupam A, Mittal B (2012). Skeletal maturation evaluation using mandibular second molar calcification stages. Angle Orthod.

[30] Różyło-Kalinowska I, Kolasa-Rączka A, Kalinowski P (2011). Relationship between dental age according to Demirjian and cervical vertebrae maturity in Polish children. Eur J Orthod.

[31] Sukhia RH, Fida M (2010). Correlation among chronologic age, skeletal maturity, and dental age. World J Orthod.

[32] Nestman TS, Marshall SD, Qian F, Holton N, Franciscus RG, Southard TE (2011). Cervical vertebrae maturation method morphologic criteria: poor reproducibility. Am J Orthod Dentofacial Orthop.

[33] Perinetti G, Di Lenarda R, Contardo L (2013). Diagnostic performance of combined canine and second molar maturity for identification of growth phase. Prog Orthod.

[34] Perinetti G, Franchi L, Castaldo A, Contardo L (2012). Gingival crevicular fluid protein content and alkaline phosphatase activity in relation to pubertal growth phase. Angle Orthod.

